# A narrative review on in-flight use of consumer sleep technologies for aviation research

**DOI:** 10.1093/sleepadvances/zpaf076

**Published:** 2025-10-28

**Authors:** Jaime K Devine, Steven R Hursh

**Affiliations:** Operational Fatigue and Performance, Institutes for Behavior Resources, 2104 Maryland Ave, Baltimore, MD 21218, United States; Operational Fatigue and Performance, Institutes for Behavior Resources, 2104 Maryland Ave, Baltimore, MD 21218, United States; Department of Psychiatry and Behavioral Sciences, Johns Hopkins University School of Medicine, 608 N Wolfe St, Baltimore, MD 21205, United States

**Keywords:** shift work, consumer sleep technology, circadian rhythms

## Abstract

Aviation is a global safety-sensitive industry that employs strict guidance about the monitoring and management of fatigue. Ecological sleep data is routinely collected to assess fatigue risk in flight crew during long-haul operations for safety and regulatory purposes. There is a growing body of scientific literature that supports the evaluation and use of consumer sleep technologies (CSTs) for ecological research. CSTs have the potential to facilitate longitudinal monitoring of sleep and fatigue in the aviation context and thus improve not only the health and well-being of flight crew but the safety of their passengers as well. However, CSTs have not been robustly studied for the measurement of in-flight sleep. Flight crew regularly take in-flight rest opportunities to mitigate fatigue when the opportunity arises and it is legally permitted. Technologies that cannot accurately capture in-flight sleep are not a reliable method to use for aviation research. The goal of this narrative review is to describe how CSTs could potentially be used to collect sleep data, specifically in the in-flight environment, based on existing guidance from the scientific and regulatory literature on fatigue risk management. Aviation stakeholders and the sleep science community should work together to develop criteria for the appropriate testing and use of CSTs as part of appropriate fatigue risk management systems (FRMS).

*This article is part of the Consumer Sleep Technology Special Collection*.

**Statement of Significance**

The global aviation industry relies on scientific guidance to inform decision-making about appropriate methods and technologies to use for sleep measurement. Consumer sleep technologies (CSTs) could revolutionize sleep data collection methods for fatigue risk management. In-flight sleep is a common fatigue mitigation strategy for flight crew, so the ability of CSTs to accurately measure sleep during flights is crucial. This narrative review presents the case for a need to evaluate CSTs specifically for the measurement of in-flight sleep data before they can be considered acceptable for use in aviation research.

## Introduction

Recently, in an unprecedented large-scale study of the impact of travel on sleep, Willoughby *et al.* [1] analyzed 1.5 million nights of data from travelers wearing a brand of consumer sleep technology (CST), the Oura Ring, collected from nearly 65 thousand journeys around the world. An editorial response to Willoughby *et al.* [1] published by Dr. Olivia Walch in SLEEP praises the team for their efforts, but also points out how this study advances the state of ecological sleep measurement technology [2]. Both Willoughby *et al.* [1] and Walch [2] focus on the impact of these technologies for passengers’ well-being in their publications. Passenger wellbeing is important, but the ability to accurately track sleep and activity patterns in career long-haul flight crew, who routinely and repeatedly cross multiple time zones in combination with shiftwork schedules [3, 4], would not only help scientists understand how to protect workers’ health but also how to improve global aviation safety. Sleeping on an airplane is a common enough practice for passengers, but augmented flight crew rely on in-flight rest opportunities to maintain alertness in cases where crew are flying for extended periods of time, across multiple time zones, or when they may be experiencing circadian misalignment [5]. Fatigue has been identified as either the cause of or a contributing factor in several costly major aircraft accidents in the past two decades [6, 7]. It is important to consider whether CSTs can be used as part of a fatigue risk management system (FRMS) to improve aviation safety.

FRMS are used in the aviation industry to manage the potential threat to safety that occurs when flight crew, especially pilots, experience fatigue due to workload, sleep deprivation, or circadian misalignment [5]. Many aviation regulatory organizations worldwide recommend that air carriers employ an FRMS to help manage the effects of fatigue during operations [5, 8–13]. The International Civil Aviation Organization (ICAO), the United Nations agency responsible for establishing global standards and practices for international civil aviation, defines FRMS as a “data-driven means of continuously monitoring and managing fatigue-related safety risks based upon scientific principles” [14]. Effective use of an FRMS relies on scientific guidance to inform decision-making about appropriate technologies for sleep data collection.

The scientific relevancy of CSTs has improved vastly in the past 10 years, guided in part by the proactive role that sleep medicine professionals have taken to construct parameters for the proper use and evaluation of new devices [15–17]. However, guidance that is geared toward sleep data collection in a general consumer population may not transfer directly to the aviation environment. Additionally, data collected for aviation research is more frequently shared directly with regulatory authorities than is published in peer-reviewed journals, so there is also a dearth of publicly-accessible information about if/how CSTs have already been used to support FRMS data collections. It is time for the sleep science community to discuss how CSTs may be properly evaluated and used to measure in-flight sleep for the purposes of FRMS.

The goal of this narrative review is to: (1) summarize how sleep data is used to support fatigue risk management aviation research, (2) outline the advantages and disadvantages of using a CST versus traditional sleep data methods to measure in-flight sleep, and (3) describe the existing literature that provides guidance on how best a CST may be evaluated as a research tool for in-flight sleep data collection in aviation FRMS.

### Fatigue risk management systems and sleep data collection

The goal of a sleep data collection for FRMS is to ensure that the work schedule has provided crew members with sufficient opportunities to obtain high-quality sleep in terms of duration and circadian timing [5, 18, 19]. Sleep data collections frequently include periods of in-flight sleep. Commercial long-haul aircraft often have a dedicated onboard rest facility specifically for crew to use for in-flight sleep [20]. Crew members are expected to sleep in the on-board facilities if the in-flight rest period is being used to extend the flight duty period [5]. Sleep during flight duty periods as a countermeasure for fatigue is discussed in the ICAO 2016 Manual for the Oversight of Fatigue Management Approaches [5]. In-flight sleep data may also need to be collected to demonstrate the safety of flight operations to an airline’s regulator [18]. Flight duty time limitations for flight crew differ as a function of how many pilots are available onboard to fly the aircraft (allowing the spare pilot an in-flight sleep opportunity), how many time zones are being crossed during operations, and the time of day that operations are occurring [3, 5, 10]. Carriers may petition for an exemption from these flight duty time limitations if they can show that the flight routes allow enough time for crew members to sleep so that they can perform their duties safely [21].

Carriers may also petition for approval for a deviation from prescriptive limits called an Alternative Method of Compliance (AMOC). For example, the Federal Aviation Administration (FAA) may grant air carriers an AMOC to any or all of an operation that deviates from the limits, provided that the carrier can demonstrate that the proposed operation employs fatigue risk mitigations in a manner that can be “as safe as or safer than” a similar route that is in compliance with the regulation [22]. Sleep data can help demonstrate that an in-flight rest opportunity effectively reduces fatigue as a function of sleep duration, but may also consider whether the in-flight rest facility provides a conducive environment that allows for high-quality sleep [20]. Air carriers require an accurate and fieldable means of recording sleep data in order to properly demonstrate safety equivalence as part of these petitions.

### Expectations for In-flight sleep data collection for FRMS

The International Air Transport Association (IATA) is the global trade association for airlines and has outlined its expectations for sleep data collection in aviation in the document “*Common Protocol for Minimum Data Collection Variables in Aviation Operations*” [18]. The National Aeronautics and Space Administration (NASA) Fatigue Countermeasures Lab provides further guidance on best practices for in-flight sleep data collection methods using actigraphy and sleep logs in their article “*Collecting Sleep, Circadian, Fatigue, and Performance Data in Complex Operational Environments*” [19]. ICAO outlines a list of acceptable tools for monitoring sleep and fatigue in the aviation context in Appendix B of their *“Manual for the Oversight of Fatigue Management Approaches*” [5].

IATA recommends that studies collect an objective measure of sleep duration, such as actigraphy, continuously for three days prior to the flight duty period of interest, throughout the period, and for three days after conclusion of flights to track baseline and recovery sleep as well as sleep during operations [18]. The IATA guidance specifically states that actigraphy should be used with sleep and duty logs to fully interpret data for FRMS [18]. The ICAO manual lists retrospective surveys, sleep diaries, actigraphy, and polysomnography (PSG) as acceptable measure tools for sleep [5]. ICAO also states that “new ways to measure fatigue, sleep, performance, or workload are always being developed and some will be valuable tools once they have been validated for use in aviation operations.” [5] Unfortunately, to date, neither ICAO nor other aviation authorities have definitely outlined the necessary steps it would take to validate a new method of sleep measurement for use in aviation.

### The difference between traditional sleep data collection methods and CSTs

Actigraphy and sleep logs have traditionally been used to collect sleep data for FRMS, but CSTs are garnering attention for use in sleep data collection in the aviation context. Sleep diaries allow individuals to report about the timing, duration, and/or quality of their recent sleep behavior. Sleep diaries rely on subjective assessments of sleep, and so, can sometimes be biased or inaccurate relative to objective measures [18, 23, 24]. Electronic sleep diaries are preferable to paper diaries for logistic reasons, but show similar poor agreement as paper diaries when compared to an objective sleep measure like actigraphy [24]. However, sleep diaries or related questionnaires are necessary to measure subjective sleep quality [25].

Actigraphy is a medical and research tool that collects objective sleep assessment for scientific analysis [26]. Actigraphy usually refers to a wrist-worn device that is similar in appearance and function to a CST. Standard actigraphy devices estimate sleep using primarily activity data. Actigraphy software generally relies on hand scoring by a trained researcher or the use of publicly available scoring algorithms, often with user-adjustable settings, for sleep–wake determination.

In contrast, modern CSTs may incorporate heart rate data, light exposure, pulse rate, respiration data, and/or body temperature along with activity data to determine sleep [27]. Sleep–wake determination performed by CSTs usually relies on proprietary algorithms, meaning that researchers do not know exactly how the device uses data to determine whether the wearer is asleep or not [27, 28]. Algorithms may also be updated at the company’s discretion, which limits the ability of researchers to compare sleep behavior across data collections with the confidence that the technology is measuring sleep in the same way between studies.

CSTs are designed to score sleep automatically through connected digital platforms and provide feedback directly to the wearer rather than importing the data from the device into specialized scoring software system that allows a trained researcher to analyze sleep patterns from raw data post-hoc [26, 29, 30]. The umbrella term “CST”, moreover, encompasses a wide range of consumer products that include wearables like smart watches or rings, but can also refer to smartphone applications (apps), bedroom monitors, portable electroencephalography (EEG) headbands, or other technologies [27, 31].

### Potential advantages and disadvantages to using a CST versus actigraphy for in-flight measurement of sleep

In the context of aviation, devices that can be continuously worn and capture sleep in a variety of locations make logistical sense. Long-haul flight crew sleep in a variety of locations (including their own home, ground rest facilities, onboard rest facilities, and hotels) and engage in irregular sleep patterns in response to fatigue, jet lag, social time cues, or work schedules [5, 32–35]. Continuously-worn devices are well-suited to capturing sleep across all times and places in a manner consistent with existing recommendations for data collection. Even if we only compare between continuously-worn sleep measurement devices (i.e. traditional actigraphy and wearable CSTs), they differ as a means of data collection.

The most glaring departure from actigraphy as a research measure is that CSTs grant the individual wearer access to their own data in real time. The fact that CSTs provide users with direct feedback about their sleep in real time can be considered both an advantage and a disadvantage compared to traditional actigraphy [36]. On the one hand, real-time feedback means that CSTs can be used to increase sleep awareness and provide sleep hygiene interventions [36, 37]. Real-time access to sleep data also allows users to see for themselves whether the device is accurately measuring sleep, and could drive CST manufacturers to improve device accuracy in response to consumer demand [38]. Feedback from CSTs can also be combined with suggestions on how to improve sleep, which could help users proactively manage fatigue moving forward.

Real-time feedback may also improve data integrity by allowing users to confirm or correct the duration and timing of sleep events. Many CSTs on the market today allow users to edit or add sleep events manually [39]. CSTs may allow users to edit automatically-identified sleep events by adjusting the bed or wake times. Some devices additionally allow users to add a sleep event during a time period that was not identified by the algorithm. For example, if the user takes a nap that the device does not automatically record, many CSTs, including Fitbit, Garmin, and WHOOP devices [40–42], will allow the user to enter a time period as a sleep event. The algorithm will then examine that period of activity to determine total sleep time. This is different from the tag feature in the Oura Ring, which allows a user to make a note indicating that they took a nap, but does not result in any additional sleep scoring [43].

Participants in actigraphy studies are encouraged to use an event marker to indicate the onset and offset of sleep [19, 44]. The event marker is a button on the actigraphy device that participants are instructed to push when they start or end a sleep event. Researchers who are hand-scoring the actigraphy data at a later date can use the event marker to improve the accuracy of sleep–wake detection. It should be noted that event marker compliance and accuracy in identifying sleep events differ widely between individuals [45].

Using the manual editing feature for CSTs could serve the same purpose as event markers in actigraphy. Instead of a researcher using the event marker to improve the accuracy of hand scoring, the device algorithm can be trained to use a manually-indicated time period to apply sleep–wake determination criteria. Manual editing of CST sleep data may help or hinder data integrity, depending on the individual user. To complicate things further, without a clear way to distinguish manually-edited sleep from automatically-scored sleep events, researchers may not be able to determine how manual editing affects data integrity or if users are even editing their data at all.

A disadvantage to real-time access to data is that it can result in orthosomnia, or an obsession with achieving perfect sleep based on CST data, although the prevalence of orthosomnia is relatively low in the general population [46, 47]. Real-time feedback may also be perceived as a disadvantage since feedback may influence the individual’s behavior [30] and participants may be able to change the device settings in a way that could affect data integrity [48]. Participants’ access to real-time feedback during an FRMS data collection could alter their behavior in a way that falsely reflects the safety of the operation.

For example, an airline conducts a data collection to demonstrate that a flight duty period that goes over the legal limits can be conducted safely, given the pilots’ rest opportunities before, during, and after the flight. However, data is only collected from individuals who optimize their sleep behavior using feedback from the CST. The data, in this case, may suggest that the proposed flight duty period is safe, but in fact represents only the best-case scenario that pilots would not adhere to without coaching from the CST. The data would therefore not accurately represent the fatigue risk associated with the proposed extended flight duty period.

Another important issue to consider for in-flight sleep data collection is that not all CSTs capture short sleep episodes or daytime sleep events. The ability of CSTs to accurately capture very short periods of sleep would prove very useful in studies that aim to quantify instances of Controlled Rest or microsleeps. Controlled Rest refers to a short, planned nap taken by a pilot on the flight deck, which some regulators permit as a countermeasure for fatigue [49]. Controlled Rest differs from sleep opportunities in which a pilot leaves the flight deck to rest in a designated crew rest facility. Controlled Rest is also not the same as unintentional napping, or “falling asleep at the wheel”. Understandably, pilots unintentionally falling asleep, even for an extremely brief moment, while flying an aircraft, is considered a safety concern.

A 2023 report by Baines Simmons found that 75% of surveyed European pilots reported experiencing at least one microsleep (defined as a brief, uncontrolled period of sleep) while operating an aircraft in the past four weeks [50]. CST data should not be considered a reliable measure for microsleeps, as the device could falsely suggest that a pilot is awake when they are, in fact, nodding off. Conversely, CST data may miss Controlled Rest or even bunk rest opportunities that pilots took proactively to counteract fatigue.

Until recently, the ability of CSTs to record short sleep events was largely absent from device evaluation studies [17] and the use of CSTs to assess daytime sleep or naps has not been recommended for research purposes [17, 31]. Evaluation studies are beginning to test devices’ ability to record short sleep events and naps [17, 51, 52], but there is still only limited evidence that CSTs are reliable for sleep estimation outside a major consolidated sleep event [30]. Devices may employ different thresholds for minimum sleep duration that could affect their ability to record naps [17]. For example, the Fitbit devices track sleep events that are longer than 60 minutes, but Oura’s sleep algorithm can detect naps for episodes with sleep time as short as 15 minutes [17]. Some CSTs allow users to manually start and end sleep periods, add naps, or edit sleep periods later [39]. Allowing users to confirm the accuracy of their CST-measured sleep data may improve data integrity, particularly when attempting to capture short sleep periods like naps. However, this relies on the user accurately using their device’s manual editing feature.

Some CSTs may allow researchers access to the raw data so that they may identify sleep–wake periods *post hoc* using open-source algorithms, hand scoring, or other research methodology*,* but other CSTs may only provide summary data that has been scored via proprietary algorithms. In the latter case, researchers may not be able to identify whether the device missed any sleep episodes unless there is supporting data like participant sleep logs.

Travel across time zones is another potential disadvantage for the use of CSTs in aviation. Sudden changes in clock time are known to pose a challenge for tracking circadian measures [30]. The ability of CSTs to measure sleep during time zone crossings as part of their data flow pathway is important for the measurement of real-world sleep in the aviation context, but has not received much attention as a potential limitation of CSTs’ research applicability.

Many aviation regulators have policies that state that wireless communication capabilities from personal electronics should be disabled to prevent interference with aircraft systems while on a flight [53–55]. Without wireless connectivity, CSTs can still record sleep events, but would not be able to score sleep in the cloud or on a mobile app interface. If the CST mobile app automatically adjusts to display sleep data in local time when there is wireless connectivity, the timing of an in-flight sleep event may be recorded or displayed incorrectly in the app interface. Because long-haul air crew cross time zones so frequently for short periods of time, long-haul flight crew may opt to keep track of time using either Coordinated Universal Time (UTC) or their home base time, regardless of their location, in an attempt to minimize circadian misalignment [56–58]. Keeping devices in a single time zone could help improve data integrity, but flight crew would need to change their settings manually to stop devices from automatically syncing to a new time zone.

CSTs that include global positioning system (GPS) location data may have an advantage in identifying periods of travel across time zones. Rahimi-Eichi *et al.* [59] recently used GPS data from mobile phones, in combination with actigraphy, to exclude any in-flight sleep episodes from their analysis on the premise that “actigraphy-based sleep detection is not appropriate for measuring sleep when an individual is on a shaking platform such as a plane, train, or bus”. Willoughby *et al.* [1], in contrast, used GPS data to identify and specifically report instances of in-flight sleep in their analysis of Oura ring data [1]. Willoughby *et al.* [1] found that in-flight sleep was significantly shorter and of lower quality than habitual sleep. The authors offer a few suggestions as to why in-flight sleep may be poor quality, but do not include device inaccuracy as a potential reason [1].

A disadvantage to collecting data using either actigraphy or CSTs for in-flight sleep is the aircraft itself. The motion of the aircraft, particularly when there is turbulence, may mimic human activity. A 2005 comparison of in-flight actigraphy using the Actiwatch (Mini Mitter Co., Inc., Bend, OR) did not find an effect of aircraft movement on sleep data quality compared to PSG [60]. The raw accelerometry data collected by actigraphs and CSTs is often submitted to processing that would filter out high and low frequency vibrations [30]. However, it is possible that aircraft vibrations that fall within the detection range of the device could be misinterpreted as physical activity.

Without access to the raw accelerometry data, knowledge about the specific flight’s turbulence, or knowing how accurate a device is at measuring in-flight sleep, it may be impossible to say whether the wearer’s sleep was fragmented due to a low-quality sleep environment or an imprecise measurement of their sleep. In this context, the ideal CST for in-flight sleep data collection could use GPS data to identify sleep episodes during air travel and apply data-filtering parameters that are specifically designed for sleep detection onboard an aircraft. As of the writing of this review, no CST has publicly advertised such a feature.

Access to data (raw or summarized) could constitute either an advantage or disadvantage to the user of CSTs relative to traditional actigraphy. On the one hand, traditional actigraphy generally relies on a wired connection between the actigraph and the scoring software, a process that is slow and cumbersome in contrast to retrieving data through a wireless or cloud-based platform [30]. Downloading actigraphy data requires physically collecting the actigraph, which may be additionally inconvenient when the participant is a pilot who travels frequently for work. Many CSTs utilize cloud-based services to extract and score data, which would simplify data extraction procedures because researchers could access the data regardless of the participant’s location. On the other hand, there is no clear pathway by which an independent researcher can access data from multiple users [30]. Researchers may need to partner with a data extraction service like Fitabase (https://www.fitabase.com/) or request access to data from the CST manufacturer directly in order to conduct their analyses.

Another disadvantage to CSTs for aviation research is the question of data ownership. Traditional actigraphs are typically purchased outright by a researcher; data is extracted via a wired connection to a desktop computer. Data access is outlined in a protocol that has passed an ethical review. CSTs, in contrast, introduces a third-party player, the device manufacturer, into the relationship between participant and researcher [61]. Use of the CST implies consent through the user agreement, but may not fully describe who retains the rights to access, control, share, or sell access to the data once it is collected. This question about data ownership opens up a litany of concerns about privacy protections, data security, and ethical data management. Government regulations such as the European Union’s General Data Protection Regulation (GDPR) or the United States’ Electronic Communications Privacy Act (ECPA) are designed to protect users’ privacy and personal information [62, 63]. The rate at which regulations are updated may not keep pace with the rate of technological advancements, however, which could leave users open to unforeseen areas of privacy risk [63]. Ethical, privacy, and ownership concerns about using CSTs for aviation research represent a distinct issue that should be addressed independently from the ability of the devices to accurately measure sleep.

### Current guidance on conducting a performance evaluation for CSTs

As mentioned above, the technical capabilities of CSTs are currently advancing at a rapid rate [27]. This means that the scientific literature needs to be frequently updated to account for technological advancements and new applications for CST use, including within the operational environment. Determining what constitutes appropriate CSTs for sleep tracking in aviation research depends on the scientific evaluation of these devices. Several recent publications outline the proper evaluation and use of CSTs in research settings [15, 16, 28, 30, 31, 36, 48, 64, 65]. In this section, we will summarize guidance from these publications that is relevant to the measurement of in-flight sleep.

Firstly, it is important to discuss the semantics of the term “validation”. A 2022 special article published in the Journal of *Sleep Health* describes a rigorous and standardized rationale for the evaluation of new sleep technologies, including a template for how to present results in an academic publication [15]. The 2022 *Sleep Health* special article also put forth a call to refer to studies that compare the accuracy of CSTs against another measure as “performance evaluations” rather than “validation studies”, since the word “validation” implies that the two measurements are in agreement [15].

This distinction is relevant to the applicability of CSTs for use in FRMS because the ICAO manual clearly states that new ways to measure sleep can be valuable tools “once they have been validated for use in aviation operations.” [5] There is a difference between validation testing, which refers to the act of comparing measurements between two devices, and the concept of validity, or the idea that the device’s measurements reflect the factual truth. “Validation” should not simply refer to the action of testing a CST against another measure of sleep but should meet standard criteria for accuracy. Standard criteria that CSTs must meet in order to be considered valid for use in aviation research have not yet been established.

In 2021, Menghini *et al.* [16] developed a standardized framework for testing the performance of sleep-tracking technology. The framework provides step-by-step analytic procedures, an illustrative example, and an open-source set of R functions [16]. Although the aviation context is not mentioned in this paper, the framework is designed to be flexible enough to apply to a variety of different testing circumstances [16]. Therefore, the framework established by Menghini *et al.* [16] could be used to guide an in-flight evaluation of CSTs against a f measure like PSG, actigraphy, or sleep diaries.

A 2024 state-of-the-science review by de Zambotti *et al.* [30] dedicates a paragraph to the issue of how CSTs incorporate clock time data and deal with time zone changes. The authors state that sudden changes in time zone pose a challenge to collecting quality data and recommend that researchers make sure that the devices’ “temporal coordinates are synchronized with those recorded by other data sources” [30]. Similarly, the World Sleep Society released a set of recommendations in 2025 on the use of wearable consumer sleep trackers, which stated that travel across time zones can interfere with the correct timing of sleep periods [66]. The authors state that device output is less reliable during travel and should be dealt with on a case-by-case basis. The World Sleep Society recommends that CST evaluations for industrial use should arise from a collaboration between sleep scientists and industry stakeholders [66]—in this case, FRMS stakeholders.

### Study design and statistical testing in the aviation context

The study design for a performance evaluation should be specific to the target population and operational conditions [31]. For a performance evaluation under the conditions of in-flight sleep, devices should be under the conditions where device accuracy may be diminished during in-flight sleep events, such as time zone crossings, periods of turbulence or other aircraft background motion, during short sleep episodes (i.e. naps), and under conditions of irregular sleep behavior (e.g. daytime sleep, split sleep, inconsistent sleep schedules). The sample population should be representative of the global aviation community. Sample selection should be informed by study design, with a sufficient sample population to power statistical analysis; convenience sampling is considered acceptable under certain circumstances [31]. Convenience sampling is the go-to approach for aviation research because it is nearly impossible to collect a truly random sample of pilots or other aviation professionals [67]. For studies conducted in the aviation context, researchers should obtain data from as many samples as possible and from across a variety of sources to order to provide a more robust picture of the general aviation population [67]. Researchers should also be sure to report effect sizes [67].

Current guidance on CST performance evaluation analysis recommends reporting basic mean comparison statistics (e.g. Student’s *t* test), discrepancy analysis, epoch-by-epoch analysis, when possible, and Bland–Altman plots, among other appropriate statistical tests for comparison between the CST and a reference measure of sleep [15, 16, 31]. Reifman *et al.* [68] developed a testing framework that adapts the Bland–Altman method to quantitatively determine whether a CST yields operationally acceptable estimates of sleep measures for FRMS purposes through the use of biomathematical modeling. Reifman *et al.* propose that if differences between PSG-measured sleep duration and CST-measured sleep duration resulted in mean differences that were smaller than the within-subject variability, then the CST can be considered an acceptable alternative technology. This methodology may be applicable to future performance evaluations of CSTs, but has only been tested using laboratory data to date [68].

Additional guidance on statistical analysis can be adapted from the AMOC requirement that carriers demonstrate that an alternative method is “as safe or safer than” the standard method of compliance [22]. The test recommended for determining whether an AMOC is equal to or better than that the standard is statistical non-inferiority analysis [69]. Lamp *et al.* [69] outlines how to use statistical non-inferiority to evaluate whether the difference in four measures of safety performance, including in-flight sleep, was equivalent between a non-compliant AMOC flight route and a comparison flight that was within FAA limits. Lamp *et al.* [69] compared in-flight sleep duration between the AMOC flight and the standard flight using the same method (actigraphy and logbook) rather than comparing sleep duration between two different technologies.

Equivalence testing is a subset of non-inferiority analysis that is recommended for comparative analyses in support of aviation policy decision-making [70]. Both Bland–Altman plots and non-inferiority focus on agreement between two measures rather than differences between the measures by establishing limits of agreement (LOA). Within non-inferiority testing, equivalence occurs when the mean and confidence interval (CI) of the differences between two measures fall within the LOA. Inferiority and superiority occur when the means and CI of the difference between measures extend beyond the lower LOA or upper LOA, respectively.

Equivalence testing (but not non-inferiority testing in general) could be adapted for the performance evaluation of CSTs for FRMS. In the context of CST performance evaluation, only devices that are equivalent to the reference method should be considered acceptable as a replacement technology. For example, if a CST device overestimates sleep duration relative to PSG, non-inferiority testing would incorrectly indicate that the CST was “superior” relative to PSG despite the fact that PSG is considered by many sleep researchers to be the gold standard for sleep measurement [31]. CSTs should strive to be statistically equivalent, but not “superior” (e.g. higher on a metric scale), to PSG records of sleep.

### Previous In-flight performance evaluations for the measurement of sleep

In 2005, before the advent of modern CSTs, Signal *et al.* [60] compared in-flight sleep measured by Actiwatch (Mini Mitter Co., Inc., Bend, OR) actigraphy against PSG. More recently, Devine *et al.* [71] compared a commercial actigraph, the Zulu watch (Institutes for Behavior Resources, Baltimore, MD, United States of America), against pilot sleep diaries and biomathematical predictions of sleep duration during ultra-long-range flights [71]. The study designs used by Signal et al. [60] or Devine *et al.* [71] to evaluate actigraphy performance against other measures of pilot in-flight sleep and/or biomathematical predictions could be reproduced to evaluate CSTs in the future. The results of the in-flight evaluations of actigraphy against PSG, self-report, and biomathematical modeling are included in Table 1.

**Table 1:** Summary of findings from previous in-flight evaluations of actigraphy compared to reference measures of sleep

Study	Device	Reference	Sleep metric	# observations	Device mean (SD)	Reference mean (SD)	Mean difference^†^
Signal *et al.* 2005	Actigraph (Actiwatch^‡^)	PSG	Sleep duration (minutes)	25	175 (82)	168 (89)	7
Signal *et al.* 2005	Actigraph (Actiwatch^‡^)	PSG	Sleep efficiency (%)	25	70 (21)	68 (21)	2
Signal *et al.* 2005	Actigraph (Actiwatch^‡^)	PSG	Sleep latency (minutes)	25	9 (12)	9 (7)	2
Devine *et al*. 2022	Actigraph (Zulu watch)	Biomathematical prediction (SAFTE-FAST AutoSleep)	Sleep duration (minutes)	77	246 (132)	235 (20)	11
Devine *et al.* 2022	Actigraph (Zulu watch)	Sleep diary	Sleep duration (minutes)	77	246 (132)	325 (128)	−79^*^

^*^ *p* < .01

^†^Differences are positive when the device overestimated sleep relative to the reference measure. Mean differences have been rounded to the nearest integer.

^‡^Actiwatch results reflect medium sensitivity threshold settings. For more information, see Signal *et al*. 2005 [60] or Devine *et al.* 2022 [71].

### Future applications for CSTs in aviation

Many CSTs claim to track not only sleep duration but also sleep staging [72]. There would be an obvious advantage to using a CST that can accurately estimate sleep stages during in-flight rest opportunities if the goal of the data collection is to assess the quality of the rest facility or if researchers wanted to explore the relationship between in-flight sleep architecture and other fatigue-relevant outcomes, like sleep inertia, cognitive performance, or alertness [73–75]. Previous evaluations of CST-based sleep staging compared to PSG have not been promising, but are continuing to improve [27, 30, 31, 66, 72].

Portable PSG or EEG headbands could be expected to better estimate sleep stages relative to wrist-worn devices, however, and may help researchers understand the physiology of sleep in the dedicated bunk area [20]. If the flight crew wear an EEG headband continuously in-flight, the data could also be used to explore cognitive biomarkers of mental fatigue, stress, or workload in the cockpit [76, 77]. EEG data was collected from flight crew as part of the Project Sunrise research flights [10]. However, at the writing of this review, only preliminary findings from passenger data from the research flights are available, and there is no mention as to whether the EEG data were included in the findings submitted to CASA [78].

A recent survey of aviation professionals indicated a preference for wrist-worn or finger-worn devices over smartphone apps, headbands, or other CSTs [79]. Continuously-worn CSTs that include additional biometric data like heart rate, skin temperature, or respiration may be helpful in developing biomarkers for fatigue [76, 77]. Heart rate variability, for example, has shown some potential as a biomarker of fatigue due to mental workload in the aviation context [80].

## Conclusions and Recommendations

Global aviation safety relies on diligent monitoring across a multitude of domains, including sleep and circadian science. Wearable CSTs in particular represent a promising advancement to fatigue risk management for aviation because they can provide a continuous real-world assessment of sleep across environments. However, there has not been sufficient research into how well CSTs work specifically for in-flight sleep measurement. CSTs should not be considered an appropriate technology for FRMS sleep data collection until a framework has been developed for how to properly conduct an in-flight performance evaluation that includes device comparison against a reference sleep measure [16, 31]. Based on the results of this review, we suggest the following recommendations:

1. A panel of experts in the field of aviation sleep research should be assembled to develop a performance evaluation framework and subsequent recommendations for the appropriate use of CSTs that satisfy the operational and scientific requirements of FRMS in the aviation context.
2. The performance evaluation framework should consider not only the appropriate use of statistical tests that meet scientific criteria for accuracy in comparison to a gold standard measure of sleep, but also consider the regulatory need to demonstrate equivalence between novel devices and approved data collection methods for FRMS. Variables that could affect device accuracy and/or sleep quality (e.g. turbulence, time zone crossings, etc.) should be controlled for or included as covariates in the statistical analysis.
3. Guidance for the performance evaluation of CSTs for use in aviation research should give priority to the ability of the device to reliably measure sleep metrics that are pertinent to fatigue risk management, such as sleep timing and duration, over sleep depth estimation or other physiological signals.
4. Scientific guidance on the use of CSTs should be incorporated into regulatory guidance for FRMS best practices.
5. Regulators, industry stakeholders, and sleep researchers should establish an ongoing working group to ensure that guidance for FRMS reflects any future advancements to technology and our scientific understanding of human fatigue.
